# FGF-23 facilitates osteosarcoma metastasis by modulating the miR-4463/LOXL2 axis expression via the ERK, p38, and JNK signaling pathway

**DOI:** 10.7150/ijms.118423

**Published:** 2026-01-01

**Authors:** Chun-Han Hou, Chih-Yang Lin

**Affiliations:** 1 Department of Orthopedic Surgery, National Taiwan University Hospital, No. 1, Jen-Ai Road, Taipei, 100, Taiwan.; 2 Translational Medicine Center, Shin Kong Wu Ho-Su Memorial Hospital, Taipei, 111, Taiwan.

**Keywords:** osteosarcoma, metastasis, FGF-23, LOXL2, miR-4463

## Abstract

Osteosarcoma is a highly malignant bone tumor affecting children and adolescents. Once metastasis occurs, the five-year survival rate drops to ~20%, emphasizing the need for new therapies. Fibroblast growth factor-23 (FGF-23), a bone-derived hormone, has been implicated in tumor progression, but its role in osteosarcoma remains unclear. Bioinformatics analysis using the R2 database revealed that elevated FGF-23 expression is associated with increased metastasis and reduced overall survival in osteosarcoma patients. Functional assays confirmed that FGF-23 enhances the migratory ability of osteosarcoma cells. Gene Expression Omnibus (GEO) analysis indicated that lysyl oxidase-like proteins—particularly LOXL1, LOXL2, and LOXL3—are overexpressed in osteosarcoma tissues compared to adjacent normal bone. In vitro experiments further showed that FGF-23 significantly upregulates LOXL2 expression in 143B and MG63 osteosarcoma cells, while LOXL2 knockdown via small interfering RNA (siRNA) markedly reduces cell migration. Moreover, pretreatment with ERK, p38, and JNK inhibitors or siRNAs targeting these pathways suppressed both FGF-23-induced LOXL2 expression and wound healing, indicating that FGF-23 promotes cell motility through ERK-, p38-, and JNK-dependent LOXL2 upregulation. FGF-23 stimulation also increased phosphorylation of ERK, p38, and JNK, and downregulated miR-4463. Inhibition of these pathways restored miR-4463 levels and suppressed LOXL2 expression. Taken together, these findings suggest that FGF-23 promotes osteosarcoma cell migration and may contribute to metastasis through coordinated regulation of the miR-4463/LOXL2 axis via ERK, p38, and JNK signaling. Targeting FGF-23 or its downstream signaling cascades may offer a promising therapeutic approach for metastatic osteosarcoma.

## 1. Introduction

Osteosarcoma is the most prevalent primary bone malignancy, primarily affecting adolescents and young adults 1. It commonly arises in the metaphysis of long bones, such as the distal femur and proximal tibia 2. Despite significant advances in diagnostic methods and treatment strategies, the prognosis for patients with metastatic disease remains poor 3, 4. Approximately 20%-30% of cases have metastasized at the time of diagnosis, and the 5-year survival rate of osteosarcoma patients with metastasis is about 20% 2. Therefore, understanding the molecular mechanisms driving osteosarcoma metastasis is essential for developing novel and effective treatment strategies.

Lysyl oxidase (LOX) is an extracellular copper-dependent enzyme, and recent studies have demonstrated that the lysyl oxidase protein family (including LOX, LOXL1, LOXL2, LOXL3, and LOXL4) plays a key role in the progression of osteosarcoma 5. These enzymes stabilize the extracellular matrix (ECM) and promote remodeling of the tumor microenvironment, both of which are critical for metastasis. Studies have confirmed that LOXL1 and LOXL4 are novel ZEB1-regulated genes that significantly enhance the invasive capacity of triple-negative breast cancer cells 6. LOXL2-driven ECM accumulation and fibrosis may strengthen the solid tumor microenvironment and act as a chemotactic factor that promotes cancer cell invasion and migration 7. Furthermore, LOXL2 has been shown to regulate ERK, p38, and JNK signaling and influence tumor cell proliferation in various cancers, including pancreatic and breast cancers 8. LOXL3 deficiency has been reported to produce pro-ferroptotic activity that impairs cell proliferation, migration, invasion, and epithelial-mesenchymal transition (EMT) in gastric cancer 9. These studies collectively confirm the importance of LOX family members in tumor metastasis. In pathological conditions such as osteosarcoma, excessive ECM deposition and cross-linking mediated by LOX family proteins contribute to increased tissue stiffness and tumor progression 8, 10. Overexpression of LOX family enzymes has been associated with poor clinical outcomes and reduced survival in osteosarcoma patients. In addition, WNT7B and WNT9A signaling pathways have been shown to enhance LOXL2 expression, further supporting its role in promoting osteosarcoma proliferation and metastasis 11. These findings highlight the importance of LOXL2 in osteosarcoma progression; however, the molecular mechanisms underlying its regulation remain largely unclear. Elucidating the pathways that regulate LOXL2 expression may offer a promising direction for developing targeted therapies against osteosarcoma metastasis.

MicroRNAs (miRNAs) are short, endogenous RNA molecules (18-25 nucleotides) that play crucial roles in post-transcriptional gene regulation by binding to the 3' untranslated regions (3'UTR) of target mRNAs 12. Dysregulated miRNA expression has been implicated in osteosarcoma progression, highlighting their importance in disease pathobiology. Several tumor-suppressive miRNAs, including miR-15a-5p, miR-21-5p, and miR-145-5p, have been identified as inhibitors of osteosarcoma cell proliferation and metastasis, making them potential therapeutic candidates 13-15. Moreover, fibroblast growth factor-23 (FGF-23) has been shown to regulate miRNA expression, with studies indicating that FGF-23 enhances miR-340-5p expression, thereby influencing osteosarcoma cell proliferation, migration, and invasion 16. However, the precise intermediate molecular mechanisms linking FGF-23 and miRNA regulation in osteosarcoma remain unclear. The growing body of miRNA expression data provides valuable insights into osteosarcoma metastasis and may lead to the identification of novel therapeutic targets.

FGF-23 is an endocrine fibroblast growth factor primarily secreted by osteocytes and osteoblasts, plays a pivotal role in phosphate and vitamin D metabolism 17. As a member of the endocrine FGF subfamily, alongside FGF-19 and FGF-21, FGF-23 regulates several essential cellular processes, including proliferation, migration, morphogenesis, and angiogenesis 18. FGF-23 and parathyroid hormone (PTH) jointly influence phosphate and vitamin D homeostasis, potentially affecting tumor growth and metastasis 18, 19. Recent studies have demonstrated that FGF-23 enhances prostate cancer cell proliferation and migration, suggesting its role as an oncogenic factor 20. In multiple myeloma, FGF-23 promoting angiogenesis by increasing VEGF-A expression via EGR1 activation 21. In the context of bone sarcomas, studies have demonstrated that genomic and transcriptomic characterization of undifferentiated pleomorphic sarcoma of bone (UPSb) revealed elevated FGF-23 mRNA expression compared with other sarcomas, including osteosarcoma 22. Beyond osteosarcoma, FGF-23 dysregulation has been reported in other bone-related conditions. In tumor-induced osteomalacia and familial tumoral calcinosis, abnormal FGF-23 production is central to disease pathophysiology 23. Myelodysplastic neoplasms (MDS), hematopoietic stem cell disorders characterized by ineffective hematopoiesis and bone fragility, also exhibit elevated serum FGF-23 levels in both patients and mouse models. Neutralization of FGF-23 in NUP98/HOXD13 mice improved erythropoiesis and bone mineralization, highlighting its dual regulatory role in bone and hematopoiesis 24. Interestingly, in MDS, elevated circulating FGF-23 partly originates from erythroid progenitors rather than osteocytes/osteoblasts, suggesting disease-specific regulatory mechanisms. Given its established role in multiple bone-related pathologies and malignancies, FGF-23 may also be an important driver of osteosarcoma metastasis. However, its specific molecular functions in this context remain poorly defined. Investigating the interplay between FGF-23 and LOXL2 in osteosarcoma may provide new mechanistic insights and identify potential therapeutic targets to inhibit malignant progression and improve clinical outcomes.

## 2. Materials and Methods

### 2.1. Materials

Recombinant FGF-23 protein was obtained from Prospec (TechnoGene, Ness-Ziona, Israel). Cell culture supplements were also supplied by Invitrogen (Carlsbad, CA, USA). Small interfering RNAs (siRNAs) targeting LOXL2, ERK, p38, and JNK, along with control siRNAs, were obtained from Dharmacon (Lafayette, CO, USA). The mimic miRNA for hsa-miR-4463 was custom-designed using the AllBio RNA system (AllBio, Taichung, Taiwan). All other reagents were purchased from Sigma-Aldrich (St. Louis, MO, USA).

### 2.2. Database analysis

Transcriptome profiles for osteosarcoma were obtained from The Cancer Genome Atlas (TCGA) via the Genomic Data Commons (GDC) platform, utilizing data hubs provided by UCSC Xena (https://xena.ucsc.edu/). We employed the R2: Genomics Analysis and Visualization Platform to analyze the Gene Expression Omnibus (GEO) dataset GSE42352 (https://hgserver1.amc.nl/cgi-bin/r2/main.cgi?open_page=login), with a focus on assessing the impact of FGF-23 expression on metastasis-free survival probability in osteosarcoma patients using Kaplan-Meier survival analysis. Additional gene expression data (GSE218035) were obtained from the GEO database, encompassing comprehensive gene expression profiles from 13 normal tissue samples and 23 human osteosarcoma tumor samples to investigate the expression profiles of the LOX family genes 25.

### 2.3. miRNA-target analysis

Potential microRNA (miRNA) interactions with the LOXL2 gene were predicted using the miRWalk database (http://mirwalk.umm.uni-heidelberg.de/) 26. Further integration with the miRDB database identified 23 miRNAs with potential binding affinity to LOXL2, using a minimum threshold filter of 0.9. The primer sequences used for each miRNA gene are listed in Table 1.

### 2.4. Cell cultures

The human osteosarcoma cell lines 143B and MG63 were sourced from the American Type Culture Collection (ATCC) (Manassas, VA, USA). Stable FGF-23-overexpressing cell lines (143B/FGF-23 and MG63/FGF-23) were generated based on established transfection protocols 27. All cell cultures were maintained in Dulbecco's Modified Eagle Medium (DMEM) supplemented with 10% fetal bovine serum (FBS), along with penicillin and streptomycin, and incubated at 37°C in a humidified environment containing 5% CO₂ 28.

### 2.5. Real-time quantitative polymerase chain reaction amplification

Total RNA was extracted from osteosarcoma cells using TRIzol reagent (MDBio Inc., Taipei, Taiwan) following the manufacturer's protocol 29. Reverse transcription was performed using 2 μg of RNA with oligo (dT) primers. qPCR was conducted using TaqMan® One-Step PCR Master Mix (Applied Biosystems, CA) in a 25 μL reaction containing 2 μL of cDNA template, 1 μL of specific primers, and 10 μL of SYBR Green. GAPDH was used as an internal control. The primer sequences used for each target gene are listed in Table 2. Reactions were performed in triplicate using a StepOnePlus sequence detection system. Thermal cycling conditions included 10 minutes at 95°C, followed by 40 cycles of 15 seconds at 95°C and 60 seconds at 60°C.

### 2.6. Western blot analysis

Cell lysates were prepared using RIPA buffer, and protein concentrations were determined using the BCA assay 30. Equal amounts of protein were separated via SDS-PAGE on 10% gels and transferred onto polyvinylidene difluoride (PVDF) membranes. Membranes were blocked with 4% bovine serum albumin (BSA) for 1 hour at room temperature, then incubated overnight at 4°C with primary antibodies (details provided in Table 3) at the indicated dilutions. After washing, membranes were incubated with peroxidase-conjugated secondary antibodies (1:3000 dilution) for 1 hour at room temperature. β-actin served as loading controls for normalization. Protein bands were visualized using a Fujifilm LAS-3000 imaging system (Fujifilm, Tokyo, Japan) 31.

### 2.7. Transwell cell migration assays

To assess cell migration, osteosarcoma cells were pre-treated with specific inhibitors for 30 minutes or siRNAs overnight. Migration was evaluated using 24-well Transwell plates (Costar, NY, USA) with an 8 μm pore size. Approximately 1.5 × 10^4^ osteosarcoma cells were seeded into the upper chamber in 200 μL of serum-free medium, while the lower chamber contained 300 μL of serum-free medium supplemented with FGF-23. After 18 hours of incubation at 37°C in a humidified 5% CO₂ environment, cells remaining on the upper surface of the insert were gently removed with a cotton swab. The migrated cells on the lower surface were then fixed in 3.7% formaldehyde for 15 minutes and stained with 0.05% crystal violet for another 15 minutes. Cell migration was quantified by counting the stained cells in the lower chamber under a microscope 32.

### 2.8. Wound healing assays

Wound-healing assays were performed according to the manufacturer's protocol using culture inserts (Ibidi, Munich, Germany) positioned in 12-well plates and allowed to stabilize for 1 hour. Once stabilized, 3 × 10^4^ osteosarcoma cells in 100 μL of culture medium were seeded on each side of the insert and cultured until they reached approximately 90% confluence. The insert was then carefully removed to create a cell-free gap or “scratch.” Wells were rinsed with phosphate-buffered saline (PBS) and filled with 1 mL of DMEM containing 0.5% FBS. The plates were incubated at 37°C with 5% CO₂ for 24 hours. Wound closure was observed at 0 and 24 hours under an inverted microscope, with images taken for analysis. Wound healing percentage was quantified using ImageJ software, following the formula: [(initial wound area) - (final wound area)] / (initial wound area) × 100 33.

### 2.9. Plasmid synthesis

The miRDB online database predicted that miR-4463 has two binding sites within the 3′UTR of LOXL2. Wild-type and mutant LOXL2 3′UTRs were generated based on the predicted miR-4463 target recognition seed sequences. The wild-type LOXL2 3′UTR was amplified and cloned into the pmirGLO dual-luciferase reporter vector (Promega, Madison, WI, USA) using the NheI and BglII restriction sites. All constructs were confirmed by Sanger sequencing to ensure correct insertion of the 3′UTR fragment. The mutant LOXL2 3′UTR (LOXL2-3′UTR-Mut), with substitutions in the miR-4463 seed-matching region, was synthesized and purchased from Invitrogen (Carlsbad, CA, USA).

### 2.10. Transfection and luciferase reporter assays

For the reporter assay, osteosarcoma cells were seeded in 24-well plates and co-transfected with 1 μg of either wild-type or mutant LOXL2 3′UTR reporter plasmid and miR-4463 mimic or negative control mimic using Lipofectamine 3000 (Invitrogen, USA), according to the manufacturer's protocol. A Renilla luciferase plasmid was co-transfected as an internal control for normalization. After 24 h, luciferase activity was measured using the Dual-Luciferase Reporter Assay System (Promega) on a luminometer, following the manufacturer's instructions. Firefly luciferase activity was normalized to Renilla luciferase activity.

### 2.11. Statistical analysis

Statistical analyses and graph preparation were conducted using GraphPad Prism 8.0 (GraphPad Software, San Diego, CA, USA). All experiments were independently repeated at least three times, with each sample analyzed in triplicate unless otherwise stated. Data are presented as the mean ± standard deviation (SD). Comparisons between two groups were performed using an unpaired two-tailed Student's t-test. For comparisons among multiple groups, one-way analysis of variance (ANOVA) followed by Tukey's post hoc test was applied. Kaplan-Meier survival curves were compared using the log-rank (Mantel-Cox) test. Correlations between variables were assessed using Pearson's correlation coefficient. p values < 0.05 were considered statistically significant. Outliers were identified and excluded using the ROUT method with a Q value of 1%, as implemented in GraphPad Prism.

## 3. Results

### 3.1. FGF-23 is highly expressed in metastatic osteosarcoma tissue and is involved in osteosarcoma progression

**Figure 1:**
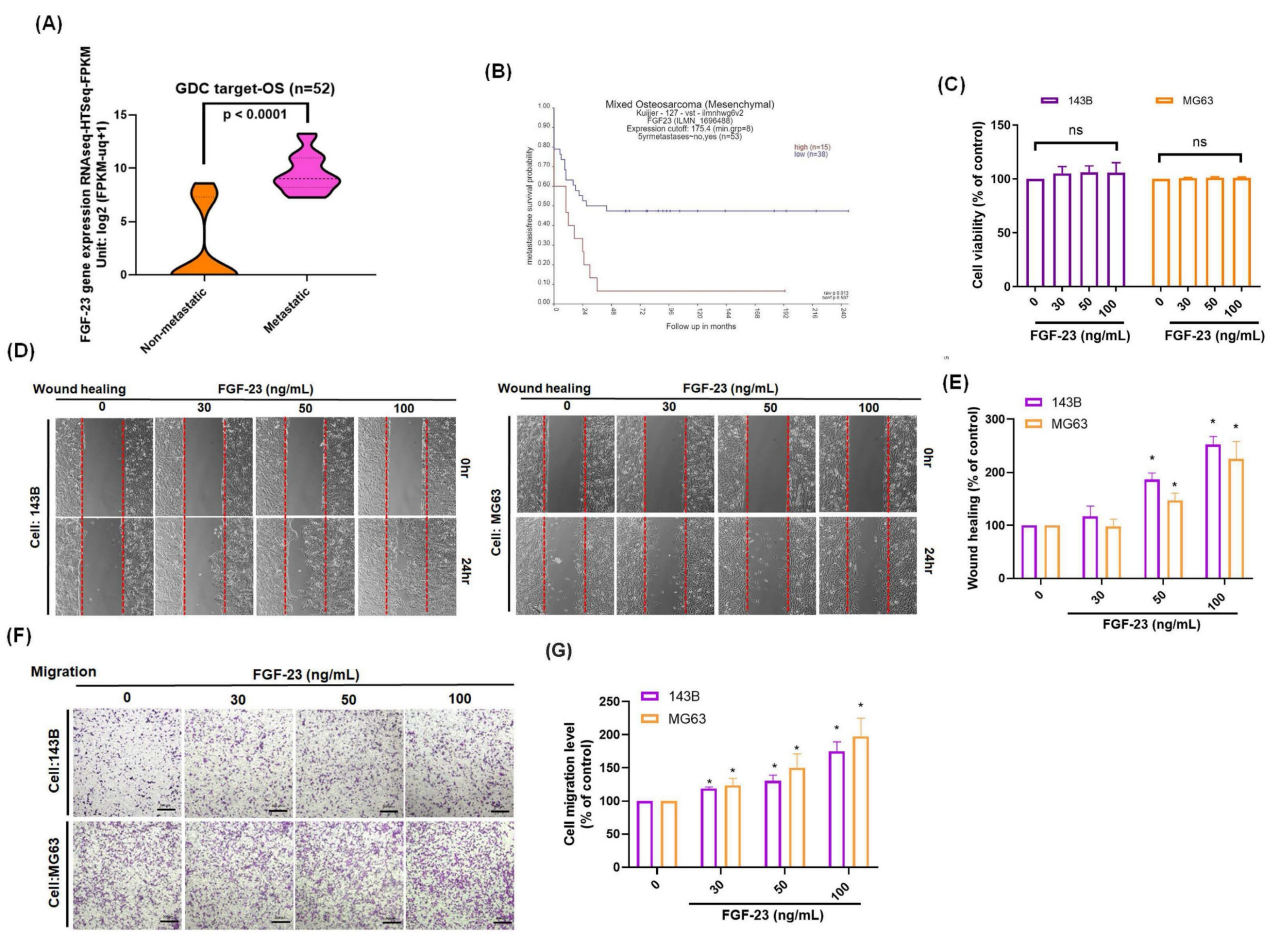
** High FGF-23 expression is associated with poor survival and increased metastatic potential in osteosarcoma.** (A) Analysis of FGF-23 gene expression in osteosarcoma tissue samples from the GDC data portal. (B) Correlation between high FGF-23 expression and metastasis-free survival probability, based on the R2 online dataset. (C) Cell viability of 143B and MG63 cells treated with FGF-23 (0-100 ng/mL) for 24 h showed no significant change. (D-G) Wound healing and Transwell assay results indicating the cell migration of osteosarcoma cells incubated with FGF-23 at various concentrations (30-100 ng/mL) for 18 or 24 h. scale bar 500 μm. All experiments were performed in triplicate and repeated at least three times. *p < 0.05 compared with the control group.

The FGF signaling pathway has been shown to play a central role in regulating the metastatic potential of osteosarcoma cells 16. Analysis of the GDC TARGET-OS cohort revealed a positive correlation between FGF-23 expression and metastatic phenotype in osteosarcoma patients (Fig. 1A). Additionally, analysis using the R2 database indicated that high FGF-23 expression was negatively associated with metastasis-free survival probability (Fig. 1B). Stimulation of osteosarcoma cells with various concentrations of FGF-23 did not affect cell viability (Fig. 1C). Wound healing and Transwell assays demonstrated a dose-dependent increase in the migratory capacity of osteosarcoma cells following FGF-23 treatment (Fig. 1D-G). Collectively, these findings suggest that FGF-23 enhances osteosarcoma cell migration in vitro and may contribute to the metastatic progression of osteosarcoma.

### 3.2. FGF-23 facilitates osteosarcoma metastasis through the upregulation of LOXL2

**Figure 2:**
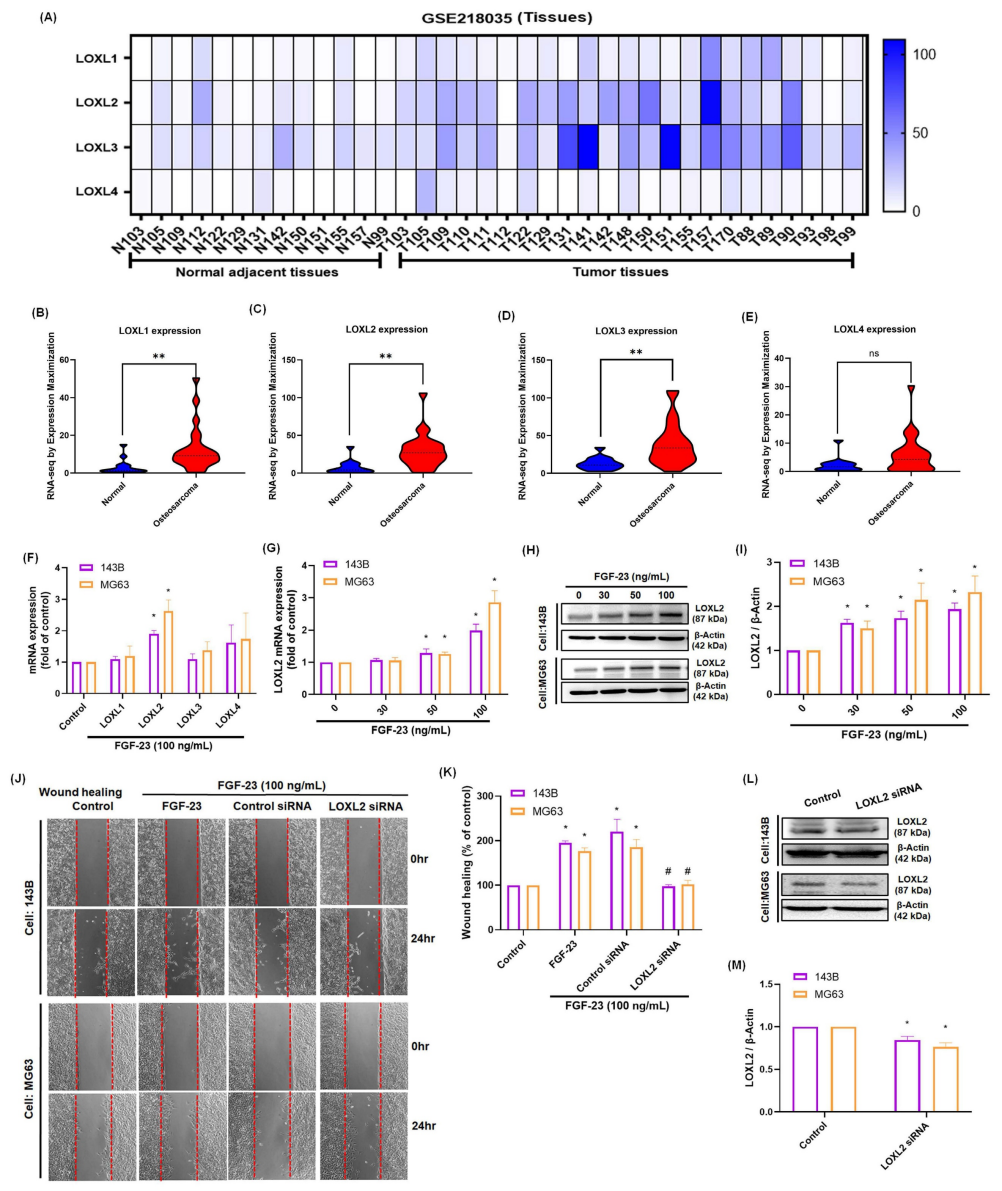
** FGF-23 enhances LOXL2 expression and promotes the migration of osteosarcoma cells.** (A-E) Analysis of LOX family mRNA expression in osteosarcoma and normal adjacent tissue in samples from the GEO GSE218035 dataset. (F) qPCR results showing the mRNA expression of four LOXL isoforms in osteosarcoma cells following incubation with FGF-23 for 24 h. (G-I) qPCR and Western blot assays showing LOXL2 expression levels in osteosarcoma cells following 24h incubation with FGF-23 at various concentrations (30, 50, or 100 ng/mL). (J&K) Wound healing showing cell migration levels in osteosarcoma cells stimulation using FGF-23 after transfection with LOXL2 siRNAs for 24 h (scale bar 500 μm). (L&M) Cells were transfected with siRNAs targeting LOXL2 for 24 hours, and protein expression was analyzed via western blotting. All experiments were performed in triplicate and repeated at least three times. *p < 0.05, **p < 0.01 compared with the control group; #p < 0.05 compared with the FGF-23-treated group.

Proteins in the LOX family play a crucial role in tumor metastasis, with LOXL2 specifically shown to enhance the invasive and migratory capabilities of various cancer cells 5, 34. Analysis of the GEO dataset (GSE218035) revealed that LOXL1, LOXL2, and LOXL3 expression levels were significantly elevated in osteosarcoma tissues compared to normal bone tissues (p < 0.01), whereas no significant difference was observed for LOXL4 expression (Fig. 2A-E). To determine whether FGF-23 regulates LOX family members, real-time PCR analysis was performed following FGF-23 treatment in 143B and MG63 osteosarcoma cells. The results showed that FGF-23 significantly increased LOXL2 expression, while no significant changes were observed for LOXL1, LOXL3, or LOXL4 (Fig. 2F). Subsequent dose-dependent assays confirmed that FGF-23 upregulated LOXL2 mRNA and protein expression as determined by qPCR and Western blot analysis (Fig. 2G-I). Wound healing assays further demonstrated that FGF-23 enhanced osteosarcoma cell migration, an effect that was significantly inhibited by LOXL2 siRNA transfection (Fig. 2J-M). Together, these findings provide strong evidence that FGF-23 promotes osteosarcoma cell migration by specifically upregulating LOXL2 expression.

### 3.3. FGF-23 induces LOXL2 expression and promotes osteosarcoma cell migration through ERK, p38, or JNK signaling pathways

**Figure 3:**
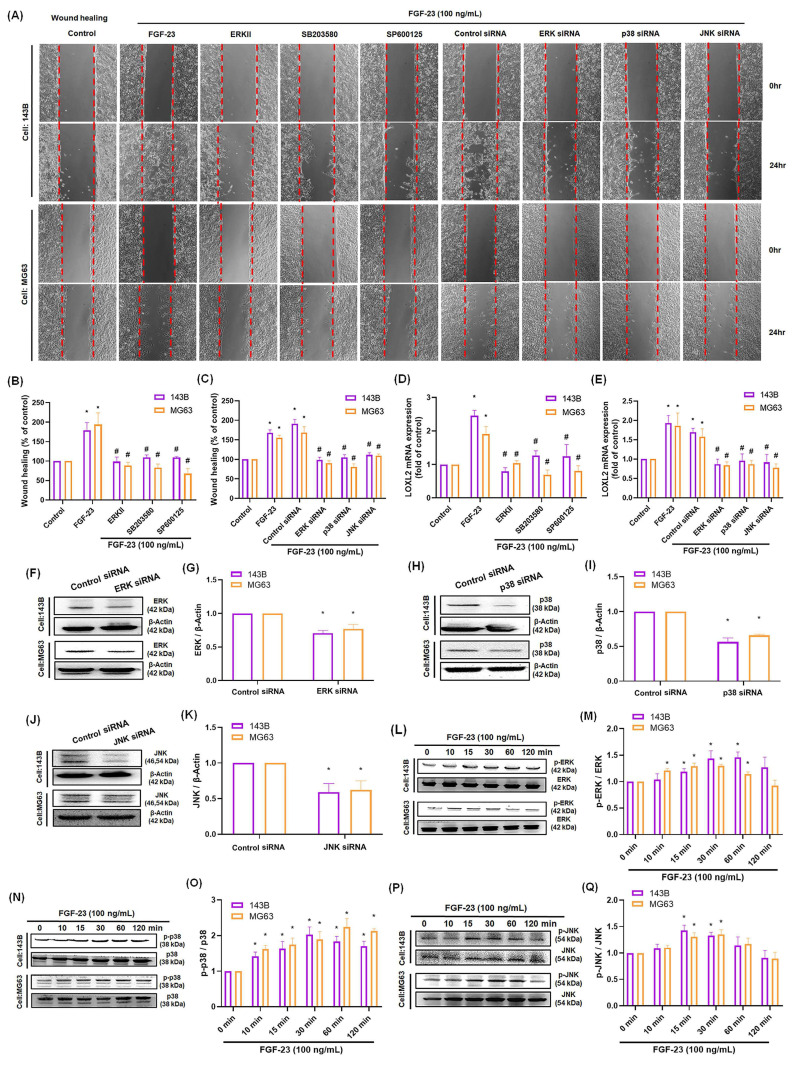
** ERK, p38, and JNK pathways contribute to FGF-23-induced LOXL2 expression and migration in osteosarcoma cells.** Osteosarcoma cells were pretreated with ERK inhibitor (1 μM), SB203580 (10 μM), or SP600125 (10 μM) for 30 minutes, or transfected with siRNAs targeting ERK, p38, or JNK for 24 hours, followed by FGF-23 stimulation. (A-C) Cell migration was assessed using wound healing assays. (D&E) LOXL2 mRNA expression levels were quantified by qPCR. (F-K) Western blot analysis shows ERK, p38, and JNK protein levels in cells transfected with the respective siRNAs. (L-Q) Time-course Western blot analysis of ERK, p38, and JNK phosphorylation in response to FGF-23 treatment (0-120 minutes). Scale bar = 500 μm. All experiments were performed in triplicate and repeated at least three times. *p < 0.05 compared with the control group; #p < 0.05 compared with the FGF-23-treated group.

Previous studies have demonstrated that activation of the ERK, p38, or JNK pathways can stimulate LOXL2 expression in various cancer cell lines 8, 35. In our study, wound healing and qPCR assays showed that pretreatment of osteosarcoma cells with ERK, p38, or JNK inhibitors—or siRNAs targeting ERK, p38, or JNK—significantly reduced FGF-23-induced cell migration and LOXL2 expression (Fig. 3A-E). Western blot analysis confirmed that transfection with siRNAs targeting ERK, p38, or JNK effectively suppressed the expression of the corresponding proteins (Fig. 3F-K). Additionally, Western blotting showed that FGF-23 stimulation increased ERK, p38, and JNK phosphorylation in osteosarcoma cells (Fig. 3L-Q). These results demonstrate that FGF-23 promotes LOXL2 expression and enhances the migratory capacity of osteosarcoma cells via ERK-, p38-, and JNK-dependent signaling. Collectively, our findings suggest that these pathways play a key role in mediating FGF-23-driven osteosarcoma metastasis.

### 3.4. The miR-4463/LOXL2 axis mediates FGF-23-induced wound healing in osteosarcoma cells

**Figure 4:**
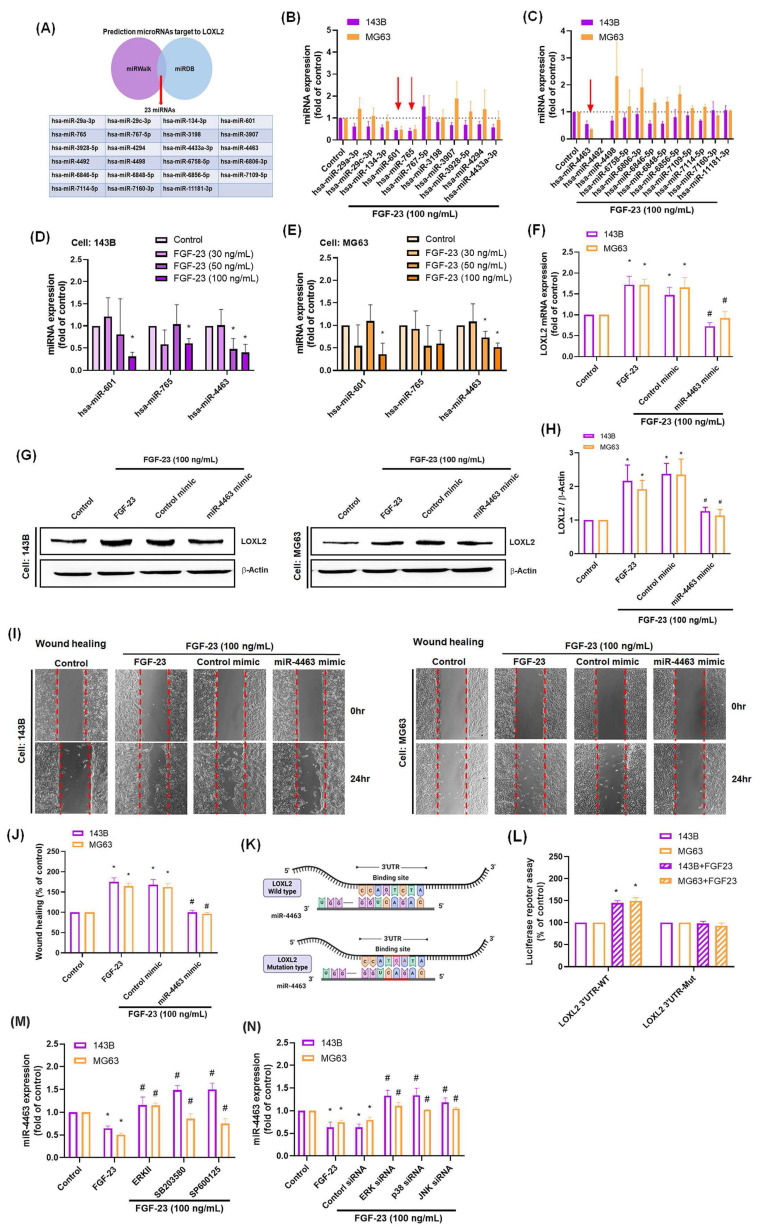
** FGF-23 suppresses miR-4463 to promote LOXL2-mediated migration in osteosarcoma cells.** (A) The miRWalk and miRDB databases jointly predicted 23 miRNAs that potentially bind to the 3′UTR of LOXL2. (B&C) qPCR analysis of 23 miRNAs expression in osteosarcoma cells following FGF-23 stimulation. (D&E) Osteosarcoma cells were treated with different concentrations of FGF-23 (30, 50, 100 ng/mL) for 24 h, and miRNA expression was examined by qPCR. (F-J) qPCR, Western blot, and wound healing assays showing LOXL2 expression and cell migration in osteosarcoma cells transfected with miR-4463 mimic or control mimic, followed by FGF-23 treatment. (K&L) Luciferase reporter assay showing relative luciferase activity in osteosarcoma cells transfected with wild-type or mutant LOXL2 3′UTR plasmids and stimulated with FGF-23 for 24 h. (M&N) Osteosarcoma cells were pretreated with ERK, p38, or JNK inhibitors for 30 min, or transfected with siRNAs for 24 h, then stimulated with FGF-23 for 24 h, the miR-4463 expression was quantified by qPCR. All experiments were performed in triplicate and repeated at least three times. *p < 0.05 compared with the control group; #p < 0.05 compared with the FGF-23-treated group.

MiRNAs are known regulators of tumor biology, capable of modulating multiple genes and cellular processes such as proliferation, differentiation, metastasis, and apoptosis 36. In this study, we investigated the role of miRNAs in FGF-23-induced LOXL2 expression and osteosarcoma cell migration. Using the online databases miRWalk and miRDB, we identified 23 candidate miRNAs predicted to target LOXL2 (Fig. 4A). Among these, treatment of osteosarcoma cells with 100 ng/mL FGF-23 significantly downregulated miR-601, miR-765, and miR-4463 (Fig. 4B&C). Notably, only miR-4463 expression was suppressed by FGF-23 in a dose-dependent manner (Fig. 4D&E). Transfection with miR-4463 mimics significantly attenuated FGF-23-induced LOXL2 mRNA and protein expression, as well as cell migration (Fig. 4F-J). To confirm a direct interaction between miR-4463 and the 3′UTR of LOXL2, luciferase reporter constructs containing either wild-type or mutant 3′UTR sequences were used. FGF-23 stimulation increased luciferase activity in cells transfected with the wild-type construct but not in those with the mutant construct (Fig. 4K&L). Furthermore, pretreatment with ERK, p38, or JNK inhibitors or their corresponding siRNAs reversed the FGF-23-mediated suppression of miR-4463 expression (Fig. 4M&N). These findings suggest that FGF-23 promotes LOXL2 expression and osteosarcoma cell migration by downregulating miR-4463 through ERK, p38, and JNK signaling pathways.

### 3.5. Overexpression of FGF-23 enhances LOXL2 expression, promotes cell migration, and suppresses miR-4463 expression in osteosarcoma cells

**Figure 5:**
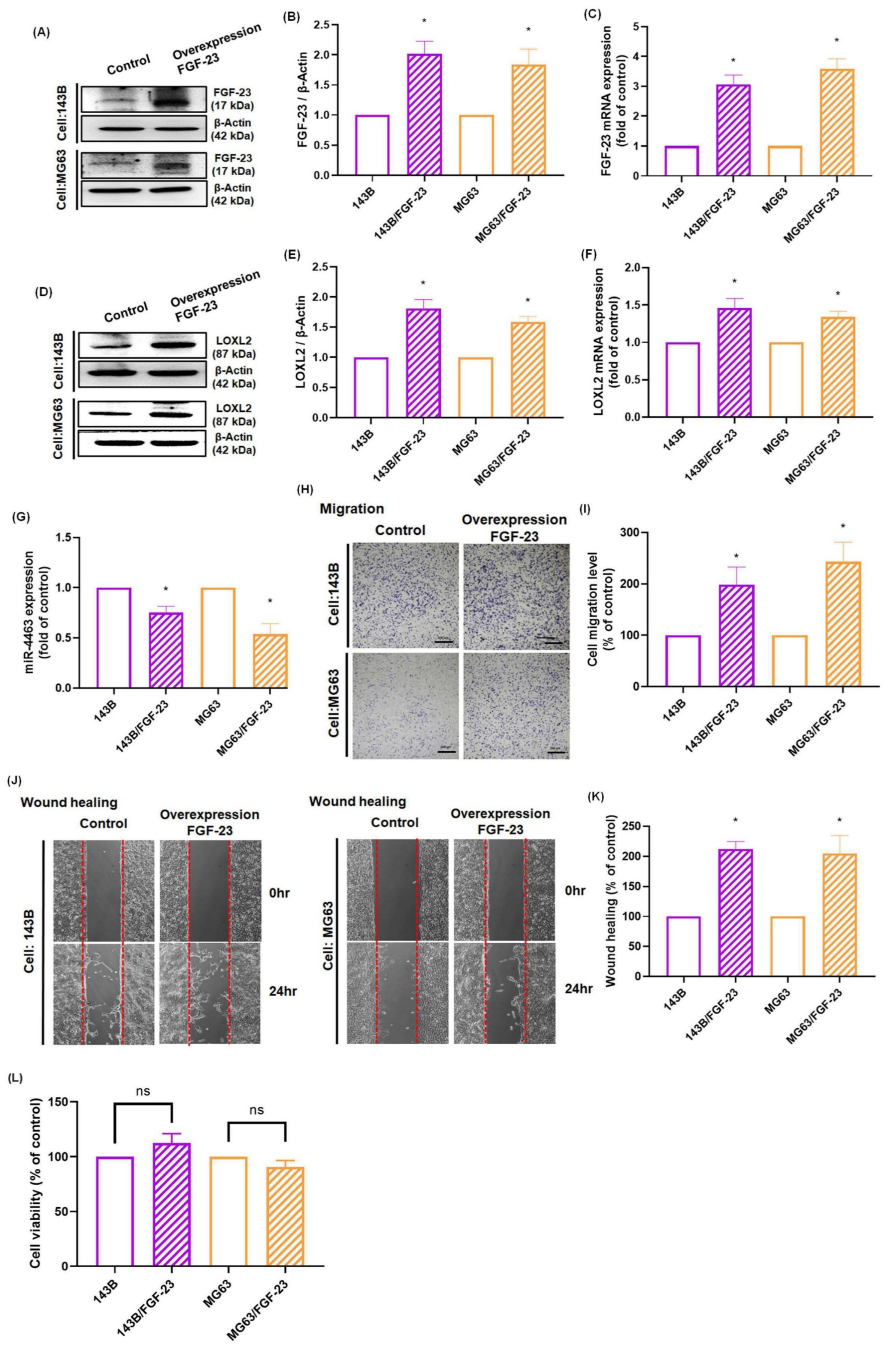
** Endogenous FGF-23 overexpression regulates the miR-4463/LOXL2 axis and enhances osteosarcoma cell migration.** (A-C) Western blot and qPCR analysis confirmed successful FGF-23 overexpression in 143B and MG63 osteosarcoma cells (143B/FGF-23 and MG63/FGF-23). (D-F) LOXL2 protein and mRNA levels were significantly elevated in FGF-23-overexpressing osteosarcoma cells. (G) qPCR analysis showed that miR-4463 expression was significantly reduced following FGF-23 overexpression. (H-K) Transwell migration and Wound healing assays revealed enhanced cell migration in FGF-23-overexpressing 143B and MG63 cells compared to control. (L) CCK8 assay confirmed that FGF-23 overexpression did not affect cell proliferation at 24 hours. All experiments were performed in triplicate and repeated at least three times. *p < 0.05 compared with the 143B or MG63 group.

Our results demonstrated that FGF-23 promotes LOXL2 expression and enhances osteosarcoma cell migration through activation of ERK, p38, and JNK signaling pathways, while simultaneously inhibiting miR-4463 expression. To further validate the functional role of FGF-23, we generated osteosarcoma cell lines (143B/FGF-23 and MG63/FGF-23) that stably overexpress FGF-23. qPCR and Western blot analyses confirmed the successful overexpression of FGF-23 and the upregulation of LOXL2 in both cell lines (Fig. 5A-F). Additionally, miR-4463 levels were significantly reduced in FGF-23-overexpressing cells compared to the control group (Fig. 5G). Functional assays using Transwell and wound healing methods revealed that FGF-23 overexpression significantly increased the migratory capacity of osteosarcoma cells without affecting cell proliferation (Fig. 5H-L). These findings demonstrate that endogenous FGF-23 promotes LOXL2 expression, suppresses miR-4463 expression, and enhances the migration of osteosarcoma cells. In summary, this study demonstrates that FGF-23 suppresses miR-4463 synthesis through the ERK, p38, and JNK signaling cascade, thereby facilitating LOXL2-dependent migration in osteosarcoma cells. These findings indicate that FGF-23 may serve as a valuable target for developing osteosarcoma therapies, with potential to limit tumor progression by targeting the FGF-23 signaling pathway to reduce osteosarcoma spread.

## 4. Discussion

Osteosarcoma is a highly malignant bone neoplasm predominantly affecting children and adolescents. It is characterized by elevated serum calcium levels, joint pain, pathological fractures, and a high likelihood of lung metastasis 37. The invasive and migratory potential of osteosarcoma cells depends largely on the bone extracellular matrix and the aggregate contribution of multiple signaling pathways and molecules 38. Fibroblast growth factor-23, a phosphaturia hormone that regulates phosphate and vitamin D homeostasis, has been implicated in osteosarcoma progression 16 and tumor-PTHrP-induced hypercalcemia as well as osteolytic lesions and metastatic spread 39. Fang et al. demonstrated that FGF-23 promotes osteosarcoma cell proliferation, migration, and invasion via downregulation of miR-340-5p, and that restoration of miR-340-5p reverses these effects 16. In this study, we sought to identify a mechanistic link between FGF-23 and the metastatic behavior of osteosarcoma cells, focusing on the miR-4463/LOXL2 axis and its effects in regulating the ERK, p38, and JNK signaling pathway. Importantly, we provide the first evidence that this axis regulates LOXL2, a key enzyme in extracellular matrix crosslinking, thereby promoting tumor cell migration and potentially contributing to metastatic niche formation. Online database analysis revealed that FGF-23 expression is significantly upregulated in metastatic osteosarcoma samples compared to primary tumors. Kaplan-Meier survival analysis demonstrated a negative correlation between FGF-23 expression levels and postoperative survival duration in osteosarcoma patients. In the present study, pretreatment of osteosarcoma cells with a miR-4463 mimic reversed FGF-23-mediated LOXL2 expression downstream of ERK, p38, and JNK signaling, and suppressed the enhanced migratory ability of osteosarcoma cells in vitro. These findings suggest that targeting the FGF-23/miR-4463/LOXL2 axis may offer an effective therapeutic strategy for inhibiting osteosarcoma metastasis.

Studies have shown that the LOX enzyme family, particularly LOXL2, plays a critical role in the invasion and migration potential of various cancer types, including osteosarcoma 40. LOXL2 promotes ECM remodeling and tumor cell dissemination by facilitating the cross-linking of collagen and elastin fibers 5. Furthermore, the bone ECM provides a supportive niche for osteosarcoma metastasis, where elevated LOXL2 expression contributes to increased matrix stiffness and enhanced cell-matrix adhesion 41. LOXL2 overexpression has also been shown to promote the growth of both primary and metastatic pancreatic ductal adenocarcinoma and to reduce survival in mouse models 10. Our findings suggest that FGF-23 promotes osteosarcoma cell migration and metastasis by upregulating LOXL2 expression via activation of the ERK, p38, and JNK signaling pathways. Analysis of the GEO database revealed that LOXL1, LOXL2, and LOXL3 expression levels were significantly elevated in osteosarcoma tissue samples compared to normal bone, indicating their involvement in disease progression. *In vitro* experiments confirmed that treatment of osteosarcoma cells with FGF-23 increased LOXL2 mRNA and protein expression in a dose-dependent manner, while the effects on LOXL1 and LOXL3 were comparatively limited. In wound healing assays, pretreatment with LOXL2 siRNA prior to FGF-23 stimulation significantly suppressed cell migration. These findings provide new mechanistic insights into how FGF-23 regulates LOXL2 expression and promotes metastatic potential in osteosarcoma.

Activation of MAPK (ERK, p38, and JNK) pathways plays a crucial role in regulating various cellular processes, including proliferation, survival, migration, and modulation of the tumor microenvironment 42. Previous studies have reported that MAPK signaling influences the metastatic potential of pancreatic and breast cancers through the activation of LOX family enzymes 7. Similarly, the EGFR/MAPK/LOX axis has been shown to promote the metastatic spread of non-small cell lung cancer 43. These findings underscore the pivotal role of MAPK pathways in tumor metastasis. In the present study, we demonstrated that the growth factor FGF-23 activates ERK, p38, and JNK signaling, leading to upregulation of LOXL2 and enhanced migratory capacity of osteosarcoma cells. Pretreatment with specific inhibitors or siRNAs targeting ERK, p38, and JNK effectively suppressed FGF-23-induced LOXL2 expression and reduced osteosarcoma cell migration. These results indicate that FGF-23 regulates LOXL2 expression by activating ERK, p38, and JNK signaling pathways. By elucidating the role of these pathways in mediating LOXL2-driven osteosarcoma metastasis, this study provides a deeper understanding of the molecular interplay underlying osteosarcoma progression.

**Figure 6:**
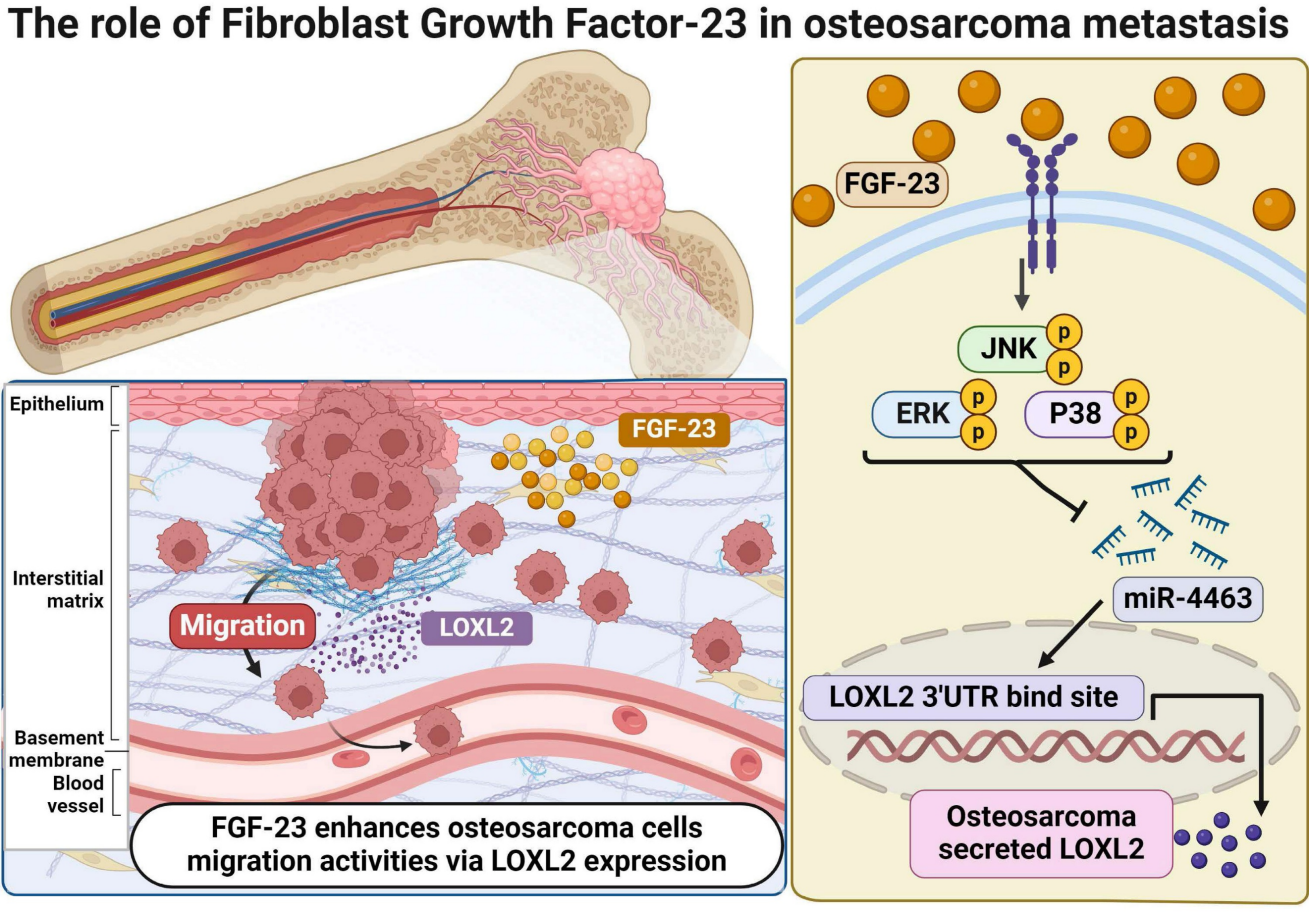
** Schematic illustration showing the process by which FGF-23 contributes to the metastasis of osteosarcoma.** FGF-23 suppresses the synthesis of miR-4463 via the ERK, p38, and JNK signaling cascade, thereby promoting the LOXL2-dependent migration of osteosarcoma cells.

MiRNAs are non-coding regulatory RNAs that function as key post-transcriptional modulators by inhibiting the translation of target mRNAs or preventing protein expression 44. Recent studies have identified 13 serum miRNAs associated with osteosarcoma progression, indicating their potential utility as diagnostic and/or prognostic markers. FGF-23 has been reported to influence the proliferation, migration, and invasion of osteosarcoma cells by promoting the expression of miR-340-5p; however, the underlying mechanisms remain unclear 16. These observations underscore the need for further investigation into the roles of FGF-23 and miRNAs in osteosarcoma. Notably, miR-4463 has also been reported to promote proliferation and migration while inhibiting apoptosis in colorectal cancer by targeting PPP1R12B, suggesting a broader oncogenic role across tumor types; our findings extend this concept by demonstrating that in osteosarcoma, FGF-23 upregulates LOXL2 via suppression of miR-4463, thereby enhancing metastatic potential 45. In this study, bioinformatics analysis identified LOXL2 as a direct target of miR-4463. Experimental validation demonstrated that FGF-23 significantly downregulates miR-4463 expression while upregulating LOXL2, thereby promoting osteosarcoma cell migration. Furthermore, pretreatment with ERK, p38, and JNK inhibitors or siRNAs reversed the FGF-23-induced suppression of miR-4463 and the increase in LOXL2 expression, indicating that the FGF-23/miR-4463/LOXL2 axis contributes to the regulation of osteosarcoma metastasis. Taken together, these findings suggest that FGF-23 exerts a pro-metastatic effect in osteosarcoma through ERK-, p38-, and JNK-mediated regulation of the miR-4463/LOXL2 axis (Fig. 6). Nevertheless, further *in vivo* studies are needed to clarify the role of this signaling axis in osteosarcoma metastasis.

In conclusion, this study demonstrates that FGF-23 promotes the migration and metastatic potential of osteosarcoma cells by upregulating LOXL2 expression through the suppression of miR-4463. Functional assays confirmed that both exogenous and endogenous FGF-23 enhance cell motility, while inhibition of key upstream signaling pathways reverses these effects. These findings highlight the significance of the FGF-23/miR-4463/LOXL2 axis in osteosarcoma progression and suggest that targeting this pathway may offer a novel therapeutic strategy to improve outcomes for patients with metastatic osteosarcoma.

### 4.1. Limitations and future directions

This study has several limitations. First, the proposed mechanism was not validated *in vivo*, and thus its physiological and translational relevance remains to be confirmed in appropriate animal models. Second, the experimental work was conducted using a limited number of osteosarcoma cell lines, which may not fully represent the genetic and phenotypic heterogeneity of the disease. Third, although our bioinformatics and in vitro data consistently support a role for FGF-23 in modulating the miR-4463/LOXL2 axis via ERK, p38, and JNK signaling, the involvement of additional pathways and regulatory molecules cannot be excluded. Future studies should incorporate comprehensive in vivo investigations, validation in a broader panel of patient-derived osteosarcoma samples, and systematic evaluation of potential crosstalk with other oncogenic signaling networks to better define the mechanistic landscape and therapeutic potential of targeting this axis in osteosarcoma.

## Figures and Tables

**Table 1:** Primer sequences used for miRNA qPCR analysis

miRNA name	Primer sequence (5'→3')
has-miR-29a-3p	TAGCACCATCTGAAATCGGTTA
has-miR-29c-3p	TAGCACCATTTGAAATCGGTTA
has-miR-134-3p	CCTGTGGGCCACCTAGTCACCAA
has-miR-601	TGGTCTAGGATTGTTGGAGGAG
has-miR-765	TGGAGGAGAAGGAAGGTGATG
has-miR-767-5p	TGCACCATGGTTGTCTGAGCATG
has-miR-3198	GTGGAGTCCTGGGGAATGGAGA
has-miR-3907	AGGTGCTCCAGGCTGGCTCACA
has-miR-3928-5p	TGAAGCTCTAAGGTTCCGCCTGC
has-miR-4294	GGGAGTCTACAGCAGGG
has-miR-4433a-3p	ACAGGAGTGGGGGTGGGACAT
has-miR-4463	GAGACTGGGGTGGGGCC
has-miR-4492	GGGGCTGGGCGCGCGCC
has-miR-4498	TGGGCTGGCAGGGCAAGTGCTG
has-miR-6758-5p	TAGAGAGGGGAAGGATGTGATGT
has-miR-6806-3p	TGAAGCTCTGACATTCCTGCAG
has-miR-6846-5p	TGGGGGCTGGATGGGGTAGAGT
has-miR-6848-5p	TGGGGGCTGGGATGGGCCATGGT
has-miR-6856-5p	AAGAGAGGAGCAGTGGTGCTGTGG
has-miR-7109-5p	CTGGGGGGAGGAGACCCTGCT
has-miR-7114-5p	TCTGTGGAGTGGGGTGCCTGT
has-miR-7160-3p	CAGGGCCCTGGCTTTAGCAGA
has-miR-11181-3p	AGGAGGAGGAGGTCAGGC

**Table 2:** Primer sequences used for qPCR analysis

Gene	Forward primer (5′→3′)	Reverse primer (5′→3′)
LOXL1	TACGATGTGCGGGTGCTAC	ATGCTGTGGTAATGCTGGTG
LOXL2	AGGATGTCGGTGTGGTGTG	TTGCGGTAGGTTGAGAGGAT
LOXL3	TGGAGTTCTATCGTGCCAATGA	CCTGAGGCTTCGACTGTTGT
LOXL4	ACTGTAGGCTGCTGGGACAC	GGTTCACAATCACCTGGAAGA
FGF-23	CAGAGCCTATCCCAATGCCTC	GGCACTGTAGATGGTCTGATGG
GAPDH	CTCCTCCACCTTTGACGC	CCACCACCCTGTTGCTGT

**Table 3:** Primary antibodies used for Western blot analysis

Target protein	Antibody name / Clone	Catalog number	Supplier	Host species	Dilution
p-ERK	E-4	SC-7383	Santa Cruz Biotechnology, CA, USA	Mouse	1:1000
ERK2	D-2	SC-1467	Santa Cruz Biotechnology, CA, USA	Mouse	1:1000
p-p38	E-1	SC-166182	Santa Cruz Biotechnology, CA, USA	Mouse	1:1000
p38	F-9	SC-271120	Santa Cruz Biotechnology, CA, USA	Mouse	1:1000
p-JNK	G-7	SC-6254	Santa Cruz Biotechnology, CA, USA	Mouse	1:1000
JNK		#9252	Cell Signaling Technology, Danvers, MA, USA	Rabbit	1:1000
LOXL2		GTX105085	GeneTex, Hsinchu, Taiwan	Rabbit	1:1000
FGF-23		PA5-121994	Invitrogen, Carlsbad, CA, USA	Rabbit	1:1000
β-actin		A5441	Sigma-Aldrich, St. Louis, MO, USA	Mouse	1:10000
